# Cascade Amplification of Pyroptosis and Apoptosis for Cancer Therapy through a Black Phosphorous-Doped Thermosensitive Hydrogel

**DOI:** 10.3390/pharmaceutics15071830

**Published:** 2023-06-26

**Authors:** Qing Wu, Qinghui Ma, Jun Ma, Junpeng Chen, Baoding Zhuang, Shanglin Yang, Jinji Liu, Shunqian Wen

**Affiliations:** 1 Department of Hepatic-Biliary-Pancreatic Surgery, Affiliated Foshan Hospital of Southern Medical University, Foshan 528000, China; 2 Department of Oncology, Affiliated Foshan Hospital of Southern Medical University, Foshan 528000, China; 3 Department of Gastroenterology, Affiliated Foshan Hospital of Southern Medical University, Foshan 528000, China

**Keywords:** cell pyroptosis, ROS, hypoxia, hydrogel, combinatory therapy

## Abstract

Cell pyroptosis has a reciprocal relationship with various cancer treatment modalities such as chemotherapy. However, the tumor microenvironment, characterized by hypoxia, substantially restricts the development and application of tumor therapies that integrate cell pyroptosis. Therefore, the cascade amplification of oxidative stress by interfering with redox homeostasis in tumors may be a promising approach. In this study, black phosphorus (BP) nanosheets and a glutathione peroxidase 4 inhibitor (RSL3) were coloaded into a thermosensitive PDLLA-PEG-PDLLA (PLEL) hydrogel (RSL3/BP@PLEL). Owing to the photothermal property of BP nanosheets, the RSL3/BP@PLEL hydrogel may trigger the release of loaded drugs in a more controllable and on-demand manner. Investigation of the antitumor effect in a mouse liver tumor model demonstrated that local injection of the hydrogel formulation in combination with near infrared laser irradiation could efficiently suppress tumor growth by interfering with the redox balance in tumors. Mechanistic study indicated that the combined treatment of photothermal therapy and glutathione depletion based on this hydrogel efficiently induced cell pyroptosis through both caspase-1/GSDMD and caspase-3/GSDME pathways, thereby triggering the repolarization of tumor-associated macrophages from M2 to M1. Overall, we developed a biocompatible and biodegradable hydrogel formulation for application in combination cancer treatment, providing a new platform for enhancing the efficacy of cancer therapy by amplifying cell pyroptosis and apoptosis.

## 1. Introduction

Cell pyroptosis, a type of proinflammatory cell death mediated by the gasdermin protein, has the potential to inhibit the development of various cancers [1,2,3,4]. Cumulative evidence also indicates a reciprocal relationship between pyroptosis and different cancer treatment modalities such as chemotherapy [5,6,7]. Mechanistically, irrespective of whether the canonical pathway (caspase-1/gasdermin D (GSDMD) dependent) or the non-canonical pathway (caspase-3/gasdermin E (GSDME) dependent) is involved, the oxidative stress induced by the excessive generation of reactive oxygen species (ROS) plays an indispensable role in the activation of cell pyroptosis [8,9,10]. However, the tumor microenvironment characterized by hypoxia considerably hinders the development and application of tumor therapies integrating cell pyroptosis [11,12,13,14]. Therefore, cascade amplification of oxidative stress by interfering with redox homeostasis in tumors may be a promising approach for overcoming the aforementioned limitation.

Thus, a strategy based on glutathione (GSH) depletion has been widely combined with cancer treatment modalities such as sonodynamic therapy, chemodynamic therapy, and ferroptosis in a synergistic manner to enhance the efficacy of cancer therapy [15,16,17,18]. Theoretically, a decrease in GSH levels in cells would significantly impair their tolerance to oxidative stress, thereby promoting the sensitivity of cancer cells to various types of cancer therapies [19,20,21]. For instance, Luo et al. reported that by targeting the gatekeeper enzyme of the pentose phosphate pathway, RRx-001 can be loaded into a hydrogel to disrupt the generation of NADPH and thereby increase ROS levels in cells. In combination with sonodynamic therapy, their approach serves as a new therapeutic platform for breast cancer [22]. With increasing research, glutathione peroxidase 4 (GPX4), the cornerstone for cell redox homeostasis maintenance, has become another intriguing target for amplifying oxidative stress in cancer cells by inhibiting the catalysis of peroxide reduction [23,24]. The antitumor efficacy of GPX4 inhibitors such as RSL3 have been observed in various cancer cell types such as fibrosarcoma, diffuse large B cell lymphomas, and colorectal cancer [25,26]. Through covalent conjugation to GPX4, inhibitors such as RSL3 may effectively induce ferroptosis in cancer cells. In addition, McManus et al. demonstrated that cotreatment with chemotherapeutic drugs and RSL3 effectively enhanced antitumor efficacy in cancer cells in a high-mesenchymal therapy-resistant cell state [27]. However, considering the lethality in adult mice with a GPX4 genetic deletion, local administration of RSL3 would be a more suitable delivery strategy until the optimal therapeutic window is determined [28]. 

Strategies to amplify ROS generation are not limited to GSH depletion. In recent years, photothermal therapy has emerged as a promising treatment modality owing to its selectivity, noninvasiveness, and biosafety [29,30,31]. By triggering photosensitizers through exogeneous stimuli, the hyperthermia induced by photosensitizers may kill cancer cells through the induction of programmed cell death such as apoptosis or pyroptosis. Moreover, accumulating evidence suggests that owing to its oxygen-independent property, photothermal therapy may ablate tumor tissues and destroy the extracellular matrix to relieve the hypoxic environment [32,33,34,35]. For instance, Zhang et al. designed and synthesized a hypoxia-induced self-assembled supramolecular complex of a perylene di-imide derivative (PDI-2CB [7]) [36]. Cucurbit [7] uril in the structure may be reduced under hypoxic conditions and transformed into a photosensitizer to achieve TME-responsive photothermal therapy in a more controllable manner. Therefore, local photothermal therapy combined with other GSH depletion strategies would be mutually beneficial for the cascade amplification of oxidative stress in tumors and for the efficient enhancement of antitumor efficacy by inducing cell pyroptosis.

**Scheme 1:**
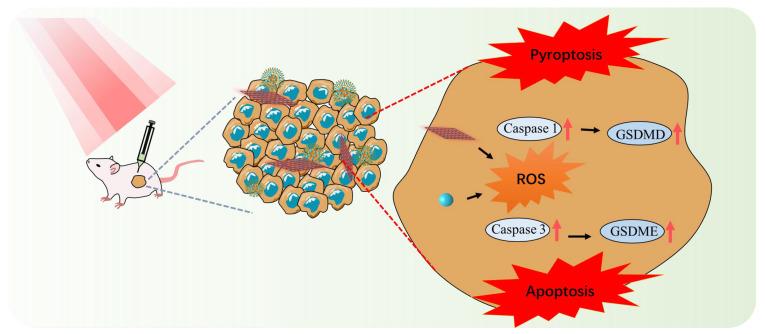
Illustration on the antitumor mechanism of the RSL3/BP@PLEL hydrogel by amplifying pyroptosis and apoptosis. Arrows represent elevated expression.

In this study, black phosphorus (BP) nanosheets and a GPX4 inhibitor (RSL3) were coloaded into a thermosensitive PLEL hydrogel (RSL3/BP@PLEL). Based on the photothermal property of the BP nanosheets, the thermal-responsive release kinetics of the RSL3/BP@PLEL hydrogel was investigated. Moreover, the antitumor efficacy and biosafety of the RSL3/BP@PLEL hydrogel were evaluated in a mouse liver tumor model bearing Hepa1-6 cells. Through histological and molecular biology analyses, we elucidated the potential antitumor mechanism of the hydrogel formulation (Scheme 1). Overall, this study, with oxidative stress as the central target, provides a new platform to enhance the efficacy of cancer therapy by amplifying cell pyroptosis.

## 2. Materials and Methods

### 2.1. Materials

PEG (*Mn* = 2000), stannous octoate (Sn(Oct)_2_), D,L-lactide (D,L-LA), bulk BP, NMP, and (1S,3R)-RSL3 (RSL3) were purchased from Sigma-Aldrich (St. Louis, MO, USA). Primary antibodies against anti-P53, anti-P21, anti-Ki67, anti-caspase-1, anti-GSDMD, anti-caspase-3, and GSDME were purchased from Abcam (Cambridge, UK). DHE, ThiolTracker™ Violet, and the TUNEL assay kit were purchased from Thermo Fisher Scientific, Waltham, MA, USA.

### 2.2. Preparation and Characterization of PLEL Hydrogel

The triblock copolymer poly(d,l-lactide)-*b*-poly(ethylene glycol)-*b*-poly(d,l-lactide) (PDLLA-PEG-PDLLA) was synthesized via stannous octoate catalyzed ring-opening polymerization. In brief, d,l-lactide and poly(ethylene glycol)_2000_ (PEG_2000_) were added to a three-necked flask under a nitrogen atmosphere. Thereafter, the catalyst stannous octoate was added at a ratio of 12,350 mol/mol (monomer/catalyst) into the flask to initiate polymerization. The mixture was stirred for 180 h at 140 °C and then cooled to room temperature. After that, the resultant was purified by dissolving in ethanol, and followed with reprecipitating from the filtrate through excess pre-cold n-pentane. Finally, the precipitates were vacuum-dried at 45 °C to obtain PDLLA-PEG-PDLLA.

The PLEL hydrogel and RSL3-loaded PLEL (RSL3@PLEL) hydrogel were prepared by self-assembly of PDLLA-PEG-PDLLA at physiologically relevant temperatures. Briefly, 20% wt PDLLA-PEG-PDLLA copolymer solution was first dispersed in PBS buffer and placed at 4 °C. Subsequently, RSL3 was added to the copolymer solution, which was then stored in a refrigerator until further use. PLEL or RSL3@PLEL was prepared in an oven at 37 °C to allow for the sol–gel transition of PDLLA-PEG-PDLLA. The morphology and structure of the as-prepared hydrogel formulations were characterized using cryo-SEM (SU 8020; HITACHI, Tokyo, Japan) and FT-IR spectroscopy (Nicolet iS 10, Shanghai, China). LCST of both hydrogel formulations was determined using MARS 60 (HAAKE, Vreden, Germany).

### 2.3. Preparation and Characterization of BP Nanosheets and RSL3/BP@PLEL

BP nanosheets were prepared according to a previously described method [37]. Briefly, the bulk black phosphorus powder was dispersed in N-methyl pyrrolidone (NMP) solution and sonicated at 1200 W for 8 h. After sonication, the exfoliated BP nanosheets were collected using centrifugation at 2000 rpm for 10 min to separate the unexfoliated nanosheets. The BP nanosheets were collected using centrifugation at 12,000 rpm for 20 min and re-dispersed in 5 mL of ethanol for storage. The exfoliated BP nanosheets were verified using TEM (FEI Tecnai F20, Columbus, OH, USA) and Raman spectroscopy (LabRam HR Evolution, Palaiseau, France). The phosphorus content of the BP nanosheets was determined by inductively coupled plasma mass spectrometry (ICP-MS). The preparation of RSL3/BP@PLEL was similar to that of RSL3@PLEL, with a pre-mixture of RSL3 (0.6 mg/mL) and BP nanosheets (100 ppm) in the copolymer solution.

### 2.4. Photothermal Property of RSL3/BP@PLEL

The photothermal property of RSL3/BP@PLEL was investigated using an 808 nm NIR (0.5 W/cm^2^) and thermal imager (HIKVISION, Hangzhou, China), both in vitro and in vivo. Briefly, PBS solution containing different concentrations of BP nanosheets (0–100 ppm) was irradiated with an NIR laser for 4 min, during which the temperature of each group was monitored and recorded for further analysis. The in vitro thermo-responsive release kinetics of RSL3/BP@PLEL was also studied. Briefly, 1 mL RSL3 @PLEL or RSL3/BP@PLEL was immersed in 9 mL PBS following with/without NIR irradiation. After that, 200 µL samples were collected at specific time intervals and another 200 µL PBS was replenished to maintain the volume. The RSL3 content in each sample was determined by high performance liquid chromatography (HPLC).

Similarly, in vivo tumor model mice were randomly divided into three groups: (1) NIR + intratumoral injection (*i.t.*) of 100 µL PBS, (2) NIR + *i.t.* injection of BP nanosheets (100 µL, 100 ppm BP nanosheets), and (3) NIR + BP@PLEL (100 µL, 100 ppm BP nanosheets). All groups were irradiated with an NIR laser for 5 min, during which the temperature of the mice was monitored and recorded.

### 2.5. Animals

All mice used in this study were purchased from Guangdong Medical Laboratory Animal Center (Guangzhou, China). All experiments were performed in accordance with a protocol approved by the Institutional Animal Care and Use Committee of the Affiliated Foshan Hospital of Southern Medical University. (Guangdong Medical Laboratory Animal Center C202211-13).

### 2.6. In Vivo Anticancer Efficacy of RSL3/BP@PLEL

The anticancer efficacy of RSL3/BP@PLEL was evaluated in the mouse liver tumor model bearing Hepa1-6 cells. The model was established by the subcutaneous (*s.c.*) injection of 2 × 10^6^ Hepa1-6 cells into nude mice. Mice with tumor volumes reaching 100 mm^3^ were selected for further evaluation. The tumor model mice were then randomly divided into six groups (5 mice per group): (1) control group (G1): *i.t.* injection of 100 µL PBS; (2) NIR + PLEL hydrogel group (G2): *i.t.* injection of PLEL hydrogel followed by 808 nm NIR irradiation (0.5 W/cm^2^, 15 min each time with a 2 min interval); (3) NIR + BP@PLEL hydrogel group (G3): *i.t.* injection of BP@PLEL hydrogel (100 µL, 100 ppm BP nanosheets) followed by 808 nm NIR irradiation (0.5 W/cm^2^, 15 min each time with a 2 min interval); (4) NIR + RSL3/PLEL hydrogel group (G4): *i.t.* injection of RSL3/PLEL hydrogel (100 µL, 3 mg/kg RSL3) followed by 808 nm NIR irradiation (0.5 W/cm^2^, 15 min each time with a 2 min interval); (5) RSL3/BP@PLEL hydrogel group (G5): *i.t.* injection of RSL3/BP@PLEL hydrogel (100 µL, equivalence of 3 mg/kg RSL3 and 100 ppm BP nanosheets); (6) NIR + RSL3/BP@PLEL hydrogel group (G6): *i.t.* injection of RSL3/BP@PLEL hydrogel group (100 µL, equivalence of 3 mg/kg RSL3 and 100 ppm BP nanosheets) followed by 808 nm NIR irradiation (0.5 W/cm^2^, 15 min each time with a 2 min interval). The entire evaluation lasted 18 days. Subsequently, all mice were euthanized, and tumors from each group were collected for H&E staining, fluorescence staining (dihydroethidium (DHE) and ThiolTracker™ Violet), TUNEL assay, immunofluorescence analysis (Ki67), and immunohistochemical analysis (P53 and P21).

Another group of tumor model mice was treated as described above and monitored for 60 days to evaluate the effect of the RSL3/BP@PLEL hydrogel on the survival rate of a Hepa1-6 xenograft mouse model

### 2.7. Pyroptosis Induced by RSL3/BP@PLEL

Pyroptosis in the tumor tissues induced by the RSL3/BP@PLEL hydrogel was confirmed using western blotting and the enzyme-linked immunosorbent assay (ELISA). The relative expression of target proteins, including caspase-1, GSDMD, GSDME, and caspase-3, was determined using western blotting as reported previously. Cytokine levels in tumor tissues indicating pyroptosis were determined using ELISA kits according to the manufacturer’s protocol.

### 2.8. Polarization of Macrophages Induced by the RSL3/BP@PLEL Hydrogel

The collected tumors were cut into 1 mm^3^ pieces using a pair of scissors. The tumor pieces were then placed on a sterile steel mesh and washed with PBS to collect the cell suspension. Lymphocytes in the suspension were obtained by adding 35% Percoll, followed by centrifugation at 600× *g* for 15 min. Thereafter, the lymphocyte suspension was further centrifuged at 1400 rpm for 5 min to collect lymphocytes and washed with 4 °C PBS two times. The lymphocytes were then labelled with the corresponding antibodies (CD45-APCY-CY7, F4/80-APC, CD86-BV510, and CD206-PE-cy7) at a dilution of 1:200 for 20 min in a refrigerator. After incubation, the lymphocytes were rinsed with PBS, centrifuged, and resuspended for flow cytometry analysis. The distribution of TAMs was analyzed using FlowJo 10.8.1 software.

### 2.9. Biodegradability and Histocompatibility of RSL3/BP@PLEL

The degradation rate of the RSL3/BP@PLEL hydrogel was first investigated in vitro for 14 days, during which the degradation rate was monitored using a UV-Vis spectrometer at specific time intervals (0, 3, 7, and 14 days). The biodegradation and histocompatibility of the RSL3/BP@PLEL hydrogel was also studied in nude mice. Briefly, a bunch of nude mice were subcutaneously administered 100 µL RSL3/BP@PLEL hydrogel and then maintained for 8 weeks; during this period, the mice were euthanized, and blood samples and major organs were collected at 6 h and 0, 2, 4, and 8 weeks post administration. The major organs, including the lung, liver, heart, spleen, and kidney, were stained with H&E to detect pathological changes. Biochemical indices, including ALT, AST, CK, and LDH levels were determined at specific time intervals using an automatic biochemical analyzer.

### 2.10. Statistical Analysis

All values are expressed as mean ± standard deviation (SD). Comparisons among the groups were performed using a one-way ANOVA or *t*-test with GraphPad Prism version 8.0, and results with *p* < 0.05, *p* < 0.01, or *p* < 0.001 were considered statistically significant.

## 3. Results and Discussion

### 3.1. Preparation and Characterization of RSL3/BP@PLEL

**Figure 1:**
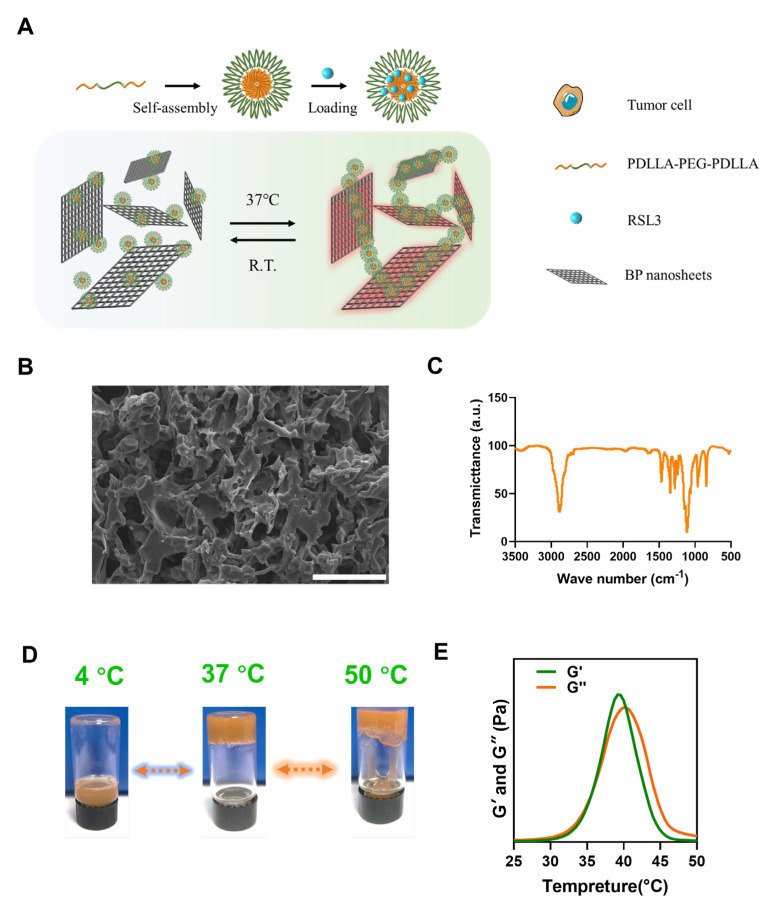
Preparation and characterization of RSL3/BP@PLEL. (**A**) Schematic illustration of RSL3/BP@PLEL. (**B**) The 3D pore structure of RSL3/BP@PLEL characterized using cryo-SEM, scale bar = 100 µm. (**C**) FT-IR spectrum of PLEL. (**D**) Sol–gel transition of RSL3/BP@PLEL under different temperatures. (**E**) Rheological analysis of RSL3/BP@PLEL.

The RSL3/BP@PLEL hydrogel was successfully prepared as depicted in Figure 1A, and gelation was achieved by exploiting the thermosensitive property of PDLLA-polyethylene glycol (PEG)-PDLLA (PLEL) at physiologically relevant temperatures. First, a thermosensitive polymer (PLEL) was synthesized via stannous octoate catalyzed ring-opening polymerization. Its hydrogel formulation was then characterized using cryogenic scanning electron microscopy (cryo-SEM), Fourier-transform infrared spectroscopy (FT-IR), and rheometric analysis. As shown in Figure 1B and Figure S1, the PLEL hydrogel presented a classic 3D structure, enabling the further loading of anticancer drugs. The structure of PLEL contains a representative carbonyl group (~1600 cm^−1^), which was detected in its FT-IR spectrum (Figure 1C). The thermosensitive property of PLEL was also verified through rheometric analysis. As shown in Figure 1D, the gelation of PLEL at 37 °C was the most efficient, whereas at 4 °C or 50 °C, it was not very efficient. The rheometric analysis results (Figure 1E and Figure S2) further confirmed the above phenomenon was because of the lower critical solution temperature (LCST; 35 °C–40 °C) of both PLEL hydrogel and RSL3/BP@PLEL hydrogel. Therefore, the thermosensitive PLEL hydrogel is suitable for further drug loading and in vivo applications.

**Figure 2:**
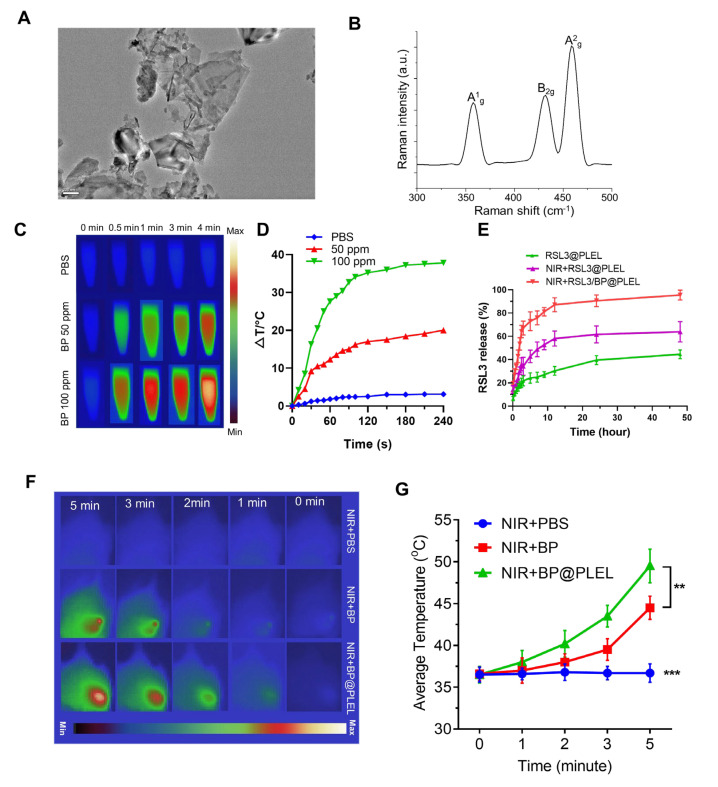
Photothermal properties of RSL3/BP@PLEL. (**A**) TEM images and (**B**) Raman spectra of BP nanosheets, scale bar = 200 nm. (**C**) In vitro photothermal images and (**D**) the corresponding temperature change in RSL3/BP@PLEL under NIR irradiation. (**E**) Photothermal responsive release kinetics of RSL3/BP@PLEL. (**F**) Photothermal images of mice bearing Hepa1-6cells after intratumoral injection of RSL3/BP@PLEL. (**G**) A plot of temperature change versus time in mice tumor. All data are shown as mean ± SD (*n* = 3), ** *p* < 0.01, and *** *p* < 0.001 vs. NIR + PBS group.

The BP nanosheets were then prepared using liquid exfoliation. Successful exfoliation of the BP nanosheets was verified using transmission electron microscopy (TEM) and Raman spectroscopy, as shown in Figure 2A,B and Figure S3. The photothermal property of the BP nanosheets, as a type of 2D material, was investigated both in vitro and in vivo. As detected using the thermal imager (Figure 2C), the temperature of the solution containing different concentrations of BP nanosheets increased with time under 808 nm laser irradiation. Moreover, according to the quantitative data (Figure 2D), compared with that of the PBS group, the temperature enhancement of 100 μg/mL BP nanosheets after 4 min of irradiation could be as high as approximately 35 °C, which enabled photothermal therapy in vivo. Considering its excellent photothermal property, the responsive drug release kinetics were also studied. As shown in Figure 2E, without laser irradiation, the cumulative release of RSL3 from the PLEL hydrogel was approximately 30%. In the RSL3/BP@PLEL group, owing to the photothermal responsiveness of the BP nanosheets, the cumulative release of the laden drugs sharply increased to approximately 80% in the first 24 h, suggesting that the release kinetics of RSL3 could be further controlled by integrating BP nanosheets into the hydrogel. Similarly, the photothermal property of BP nanosheets was also demonstrated in mice. As shown in Figure 2F,G, temperature enhancement was observed in tumor-bearing mice after the local injection of BP nanosheets and BP@PLEL followed by 5 min of irradiation. It should be noted that the temperature enhancement of the BP@PLEL group was still higher than that of the BP nanosheet group. This difference may be because the PLEL hydrogel containing water may conduct heat better, thereby exhibiting a higher temperature. Overall, the integration of photothermal responsive BP nanosheets into RSL3@PLEL hydrogel could trigger the release of loaded drugs in a more controllable and on-demand manner. Simultaneously, its excellent photothermal property ensured the effective photothermal therapy of tumors.

### 3.2. In Vivo Anticancer Efficacy of RSL3/BP@PLEL

**Figure 3:**
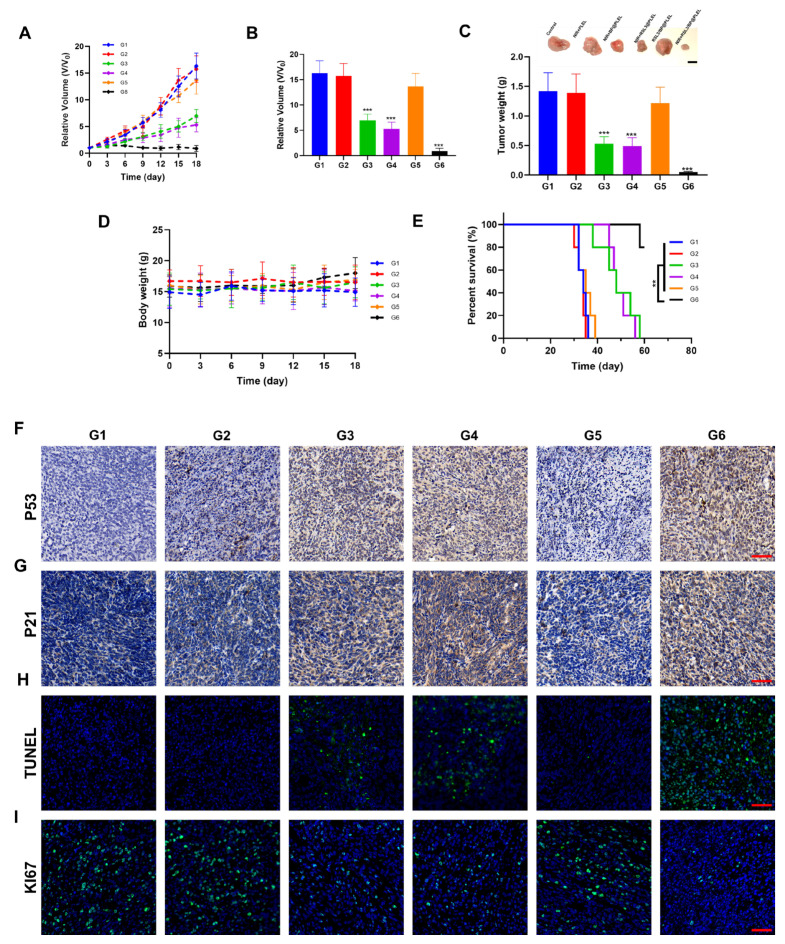
Anticancer efficacy of RSL3/BP@PLEL in mice bearing Hepa1-6 cells. A plot of (**A**) mice tumor growth, (**B**) mice tumor volume, and (**C**) mice tumor weight for each treatment group; inlet: representative photos of resected tumors collected from each treatment group, scale bar = 1 cm. (**D**) Body weight and (**E**) survival ratio of mice from each treatment group. (**F**–**I**) Histological analysis of resected tumor slices with p53, P21, TUNEL, and Ki67; scale bar = 50 µm. G1: Control group; G2: NIR + PLEL hydrogel group; G3: NIR + BP@PLEL hydrogel group; G4: NIR + RSL3/PLEL hydrogel group; G5: RSL3/BP@PLEL hydrogel group; G6: NIR + RSL3/BP@PLEL hydrogel group. All data are shown as mean ± SD (*n* = 5), ** *p* < 0.01 and *** *p* < 0.001 vs. control group.

After confirming the photothermal property and its associated responsive drug-release kinetics, we evaluated the antitumor efficacy of RSL3/BP@PLEL in vivo. As shown in Figure 3A,B, intratumoral injection of RSL3/BP@PLEL combined with near-infrared laser (NIR) irradiation significantly suppressed tumor growth, as the relative volume was approximately 1 (V/V_0_) during the 18-day evaluation period. It should be noted that although the NIR + BP@PLEL and NIR + RSL3/PLEL hydrogel groups exhibited antitumor effects, local injection of the RSL3/BP@PLEL hydrogel without NIR irradiation only slightly increased tumor growth, indicating the antitumor efficacy of photothermal therapy and its indispensable role in triggering the release of RSL3. A similar trend was observed for the resected tumor weight (Figure 3C). In comparison with the large tumors in the control group (~1.4 g), the tumor weight in the NIR + BP@PLEL and NIR + RSL3/PLEL hydrogel groups was reduced by 64.2%. In the NIR + RSL3/BP@PLEL hydrogel group, this reduction was as high as 92.8%, demonstrating the high antitumor efficacy of this hydrogel formulation. In addition to its excellent antitumor efficacy, this treatment modality is also biosafe and biocompatible, as the mouse weight during the 18 days of treatment did not fluctuate significantly (Figure 3D). The survival rate of mice in each treatment group further demonstrated this aspect. As shown in Figure 3E, in comparison with the 0% survival ratio of the control group on day 40, the NIR + RSL3/BP@PLEL hydrogel group maintained a mouse survival ratio of 100% until day 60, showing its substantial antitumor potential and prognosis.

After 18 days of evaluation, the antitumor efficacy of the NIR + RSL3/PLEL hydrogel was investigated histologically. Hematoxylin and eosin (H&E) staining of tumor slices preliminarily confirmed (Figure S4) that the purple areas, representing the nucleus of cancer cells, decreased to different extents in each treatment group, suggesting DNA damage in the tumor. Therefore, indices including P53, P21, and TUNEL were evaluated using immunofluorescence or immunohistochemical analysis. As shown in Figure 3F–I, similar trends were observed for all indices in the tumor slices. Specifically, the highest signals of P53, P21 (indicated by the brown shade), and TUNEL (indicated by the green fluorescence) were detected in the NIR + RSL3/PLEL hydrogel group, followed by the NIR + BP@PLEL and NIR + RSL3/PLEL hydrogel groups, further suggesting the antitumor efficacy of photothermal therapy and its indispensable role in triggering the release of RSL3. Correspondingly, the Ki67 level (indicated by the green fluorescence), which reflects the status of cell proliferation, significantly decreased in the NIR + RSL3/PLEL hydrogel group.

These findings demonstrate that local injection of the RSL3/PLEL hydrogel combined with NIR can inhibit tumor growth in a safe, controllable, efficient, and on-demand manner, which may be beneficial in clinical use.

### 3.3. Pyroptosis Induced by the RSL3/BP@PLEL Hydrogel

**Figure 4:**
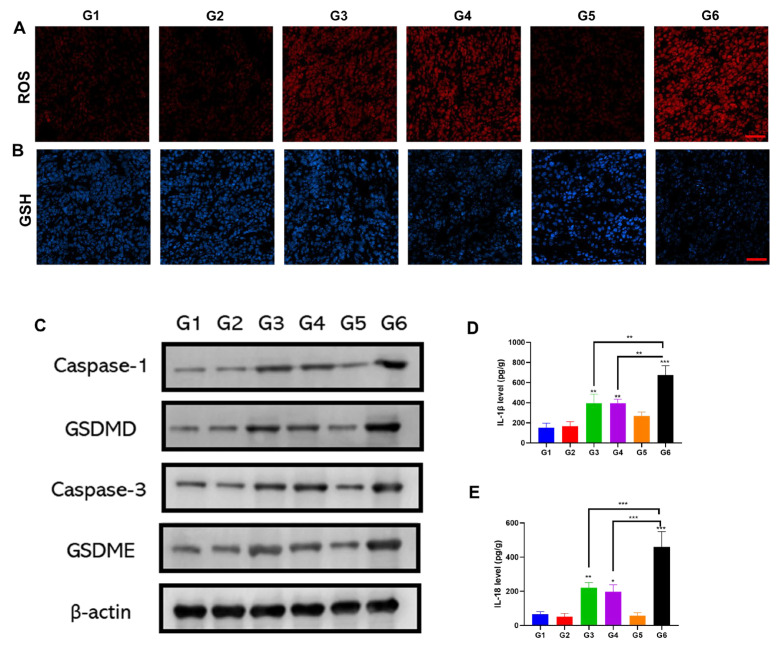
Pyroptosis induced by RSL3/BP@PLEL. (**A**) ROS level (DHE dye, red fluorescence) and (**B**) GSH level (ThiolTracker dye, blue fluorescence) in mouse tumors resected from each treatment group, scale bar = 50 µm. (**C**) Expression of caspase-1, GSDMD, caspase-3, and GSDME in tumor tissues from each treatment group using western blotting analysis. Cytokine levels, including (**D**) IL-1β and (**E**) IL-18 levels, in tumor tissues from each treatment group. G1: Control group; G2: NIR + PLEL hydrogel group; G3: NIR + BP@PLEL hydrogel group; G4: NIR + RSL3/PLEL hydrogel group; G5: RSL3/BP@PLEL hydrogel group; G6: NIR + RSL3/BP@PLEL hydrogel group. All data are shown as mean ± SD (*n* = 3), * *p* < 0.05, ** *p* < 0.01, and *** *p* < 0.001 vs. NIR + RSL3/BP@PLEL group.

Based on the excellent antitumor efficacy of the RSL3/PLEL hydrogel, its antitumor mechanism was further investigated. RSL3, an inhibitor of GPX4, has been suggested to induce cell apoptosis by triggering the overgeneration of ROS and oxidative stress. Photothermal therapy, also known as thermal ablation, can enhance the temperature of particular tumor sites under laser irradiation with the assistance of photosensitizers. Simultaneously, the overheating of particular tumor sites might enhance oxidative stress and further promote cell death. Therefore, it can be speculated that the excellent antitumor efficacy of the RSL3/PLEL hydrogel combined with NIR irradiation is mainly due to the imbalance in redox homeostasis in cancer cells triggered by both RSL3 and thermal ablation. Therefore, ROS and GSH levels in the tumor slices were evaluated. As shown in Figure 4A,B, treatment with RSL3 or thermal ablation increased ROS levels (indicated by red fluorescence) in the NIR + BP@PLEL and NIR + RSL3/PLEL hydrogel groups. Moreover, in the NIR + RSL3/BP@PLEL hydrogel group, red fluorescence was stronger, suggesting a synergistic antitumor effect of BP nanosheets and RSL3. Correspondingly, owing to the GSH-depletion effect of RSL3, in the NIR + RSL3/PLEL and NIR+ RSL3/BP@PLEL hydrogel groups, a significant decrease in GSH level was detected in the tumors. In the untreated group, the GSH level in the tumor was comparable to that of the control group.

Theoretically, local hyperthermia may induce cell death and promote the release of damage-associated molecular patterns (DAMPs), an inflammatory response in defending infection [38]. Hence, it is reasonable to deduce there exists an interplay between hyperthermia and a proinflammatory cell death pathway, i.e., pyroptosis. In addition, the amplification of ROS generation would further trigger cell apoptosis through caspase family members such as caspase-3, which may also promote cell pyroptosis through the non-canonical pathway (caspase-3/GSDME dependent). Therefore, both pathways (caspase-1/GSDMD and caspase-3/GSDME) that may trigger cell pyroptosis were investigated using western blotting. As shown in Figure 4C, in the NIR+ RSL3/BP@PLEL hydrogel group, the expression of caspase-1/GSDMD and caspase-3/GSDME was significantly upregulated. The levels of serum cytokines including interleukin (IL)-1β and IL-18 in the NIR+ RSL3/BP@PLEL hydrogel group were elevated by 2–3 fold compared with those in the control group (Figure 4D,E). Moreover, this elevation in the NIR+ RSL3/BP@PLEL hydrogel group was still 0.75–1-fold higher than that in the NIR + BP@PLEL hydrogel and NIR + RSL3/PLEL hydrogel groups. These results suggest that, on one hand, thermal ablation induces cancer cell death and enhances the immunogenicity of tumors to promote inflammatory cell death, namely cell pyroptosis. On the other hand, oxidative stress induced by thermal ablation and the GPX4 inhibitor (RSL3) promotes the expression of caspse-3 and thereby simultaneously triggers cell apoptosis and pyroptosis. Overall, the combined treatment of photothermal therapy and GSH depletion based on this hydrogel efficiently induced cell pyroptosis through both caspase-1/GSDMD and caspase-3/GSDME pathways, indicating great potential for its application in combination cancer therapy.

### 3.4. Polarization of Macrophages Induced by RSL3/BP@PLEL Hydrogel

**Figure 5:**
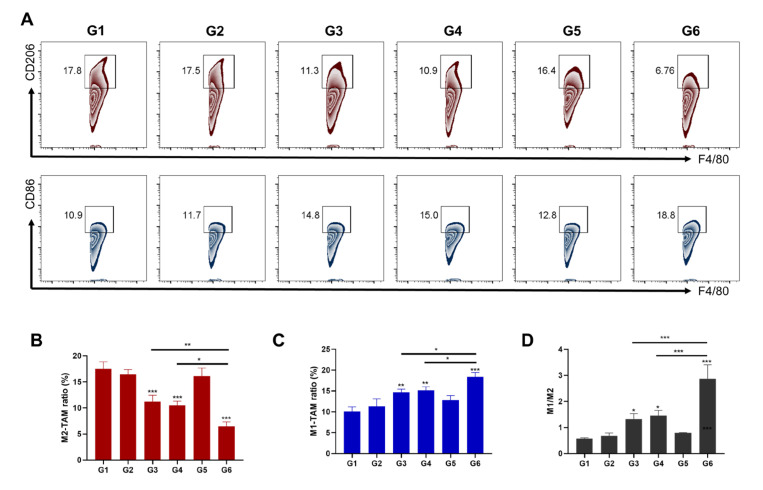
Polarization of macrophages induced by RSL3/BP@PLEL. (**A**) Flow cytometric analysis of tumor-associated macrophages from each treatment group after gating CD86 and CD206. The proportion of (**B**) M2 tumor-associated macrophages and (**C**) M1 tumor-associated macrophages in tumor tissues from each treatment group. (**D**) The ratio of M1/M2 tumor-associated macrophages in tumor tissues from each treatment group. G1: Control group; G2: NIR + PLEL hydrogel group; G3: NIR + BP@PLEL hydrogel group; G4: NIR + RSL3/PLEL hydrogel group; G5: RSL3/BP@PLEL hydrogel group; G6: NIR + RSL3/BP@PLEL hydrogel group. All data are shown as mean ± SD (*n* = 3), * *p* < 0.05, ** *p* < 0.01, and *** *p* < 0.001 vs. NIR + RSL3/BP@PLEL group.

Cell pyroptosis, as a highly inflammatory mode of cell death, may enhance the immunogenicity of tumors by promoting the secretion of cytokines such as IL-1β and IL-18. Therefore, it can be deduced that the immunosuppressive tumor environment (TME) may be alleviated by such treatment. The tumor-associated macrophage (TAM) level, an indicator of the immunosuppressive TME, was determined using flow cytometry. As shown in Figure 5A, CD206+ macrophage (M2) level was as high as approximately 17% in the control and NIR + PLEL hydrogel groups, owing to the immunosuppressive TME. As shown in Figure 5B, photothermal therapy efficiently decreased the M2 macrophage level by 36.5% in the NIR + BP@PLEL hydrogel group (11.3%), owing to tumor ablation induced by hyperthermia. Moreover, the combination therapy with RSL3 further amplified this alleviation, as the M2 macrophage level was reduced by 61.8% in the NIR + RSL3/BP@PLEL hydrogel group (6.76%). In addition, without NIR to trigger the release of RSL3 and local hyperthermia, the effect of the RSL3/BP@PLEL hydrogel on modulating macrophages was limited, as the M2 macrophage level was comparable to that in the control group. Correspondingly, as shown in Figure 5C, the CD86+ macrophage (M1) level in the NIR + RSL3/BP@PLEL hydrogel group increased to approximately 18.8% compared with that in the control group, indicating the repolarization of TAMs. In addition, monotherapy increased the number of M1 macrophages to varying extents. Remarkably, the M1/M2 ratio (Figure 5D) indicated more distinctive immunosuppressive TME conditions in each treatment group. The M1/M2 ratio of the NIR + RSL3/BP@PLEL hydrogel group was approximately 1-fold higher than that of the other treatment groups, indicating the synergistic effect of photothermal therapy and GSH depletion in inducing macrophage repolarization.

These results indicate that local photothermal therapy combined with RSL3 treatment would be mutually beneficial to the cascade amplification of cell pyroptosis in tumors and efficiently alleviate the immunosuppressive TME by inducing the re-polarization of TAMs.

### 3.5. Biodegradability and Biosafety of the RSL3/BP@PLEL Hydrogel

**Figure 6:**
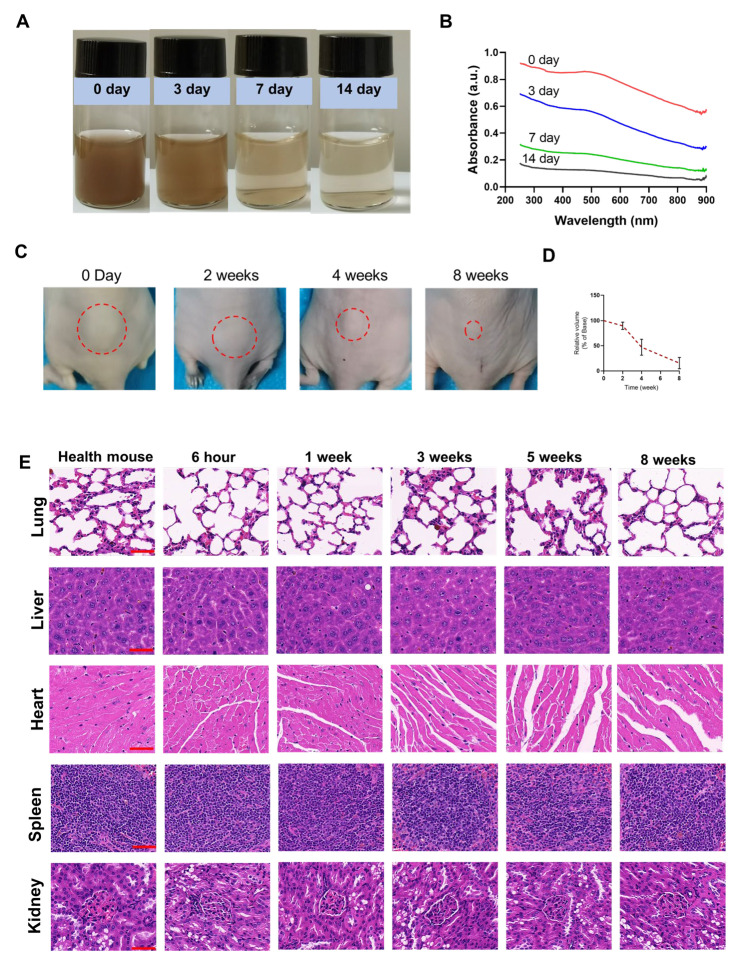
Biodegradability and biosafety of RSL3/BP@PLEL. (**A**) In vitro abiotic aging of RSL3/BP@PLEL across 14 days. (**B**) UV-Vis spectrum of RSL3/BP@PLEL after storage for 0, 3, 7, and 14 days. (**C**) In vivo biodegradability of RSL3/BP@PLEL in mice for 8 weeks. (**D**) A plot of hydrogel volume versus time after subcutaneous (*s.c.*) injection of RSL3/BP@PLEL in mice for 8 weeks. (**E**) H&E staining of the major organs of mice at 0, 6 h, 1, 3, 5, and 8 weeks post *s.c.* administration, scale bar = 50 µm. All data are shown as mean ± SD (*n* = 3).

Considering its potential clinical applications, the biodegradability and biosafety of the RSL3/BP@PLEL hydrogel was assessed. As shown in Figure 6A,B, the brownish BP nanosheets faded with an increase in time over 14 days, suggesting the degradability of the BP nanosheets. We further evaluated the biodegradability of the RSL3/BP@PLEL hydrogel. As seen in Figure 6C, shrinkage of the hydrogel (circled by red dotted lines) under the mouse skin could be distinctively observed. Quantitative data regarding the hydrogel volume (Figure 6D) further proved its biodegradability, as the hydrogel volume dropped from 100% to almost 5% after 8 weeks of evaluation. During this evaluation period, slices of major organs, including the lung, liver, heart, spleen, and kidney, did not show obvious pathological changes or inflammation. The biochemical indices (Figure S5), including alanine aminotransferase (ALT), aspartate transaminase (AST), creatine kinase (CK), and lactate dehydrogenase (LDH) levels, did not change significantly during the 8 weeks, further demonstrating the biosafety of the hydrogel.

## 4. Conclusions

In this study, a thermosensitive PLEL hydrogel loaded with BP nanosheets and a GPX4 inhibitor (RSL3/BP@PLEL) was designed and prepared. Owing to the photothermal property of the BP nanosheets, the RSL3/BP@PLEL hydrogel may trigger the release of loaded drugs in a more controllable and on-demand manner. Investigation of the antitumor effect in a mouse liver tumor model demonstrated that local injection of the hydrogel formulation in combination with NIR irradiation could efficiently suppress tumor growth by interfering with the redox balance in tumors. The mechanistic study indicated that the combined treatment of photothermal therapy and GSH depletion with this hydrogel efficiently induced cell pyroptosis through both caspase-1/GSDMD and caspase-3/GSDME pathways. Specifically, thermal ablation may enhance the immunogenicity of tumors to promote inflammatory cell death, namely, cell pyroptosis, and the amplification of oxidative stress induced by thermal ablation. RSL3 promoted the expression of caspse-3 and thereby simultaneously triggered cell apoptosis and pyroptosis. Overall, local photothermal therapy combined with the RSL3-based GSH depletion strategy is mutually beneficial to the cascade amplification of oxidative stress in tumors and efficiently enhances antitumor efficacy by inducing cell pyroptosis. In summary, we developed a biocompatible and biodegradable hydrogel platform for application in combination cancer treatment.

## Data Availability

The data presented are available from the corresponding author upon request.

## Supplementary Materials

The following supporting information can be downloaded at https://www.mdpi.com/article/10.3390/pharmaceutics15071830/s1. Figure S1: SEM image and elemental mapping of the PLEL hydrogel; Figure S2: Rheological analysis of the PLEL hydrogel; Figure S3: SEM image of BP nanosheets; Figure S4: H&E staining of tumor slices from different treatment groups; Figure S5: Biochemical indices of mice treated with RSL3/BP@PLEL at specific time intervals.
