# Supplementary material for: Cascade Amplification of Pyroptosis and Apoptosis for Cancer Therapy through a Black Phosphorous-Doped Thermosensitive Hydrogel
Source: Pharmaceutics. 2023 Jun 26;15(7):1830. doi: 10.3390/pharmaceutics15071830 (PMC10383820; doi:10.3390/pharmaceutics15071830)
Supplement: Supplementary file 1 [file pharmaceutics-15-01830-s001.zip › pharmaceutics-2406886-supplementary.pdf]

Cascade Amplification of Pyroptosis and Apoptosis for Cancer Therapy through a Black Phosphorous-Doped Thermosensitive Hydrogel

Qing Wu[1], Qinghui Ma[2], Jun Ma[3], Junpeng Chen[1], Baoding Zhuang[1], Shanglin Yang,[1] Jinji Liu[2], Shunqian Wen[1*]

[1] Department of Hepatic-biliary-pancreatic Surgery, Affiliated Foshan Hospital of Southern Medical University, Foshan 528000, China

[2] Department of Oncology, Affiliated Foshan Hospital of Southern Medical University, Foshan 528000, China

[3] Department of Gastroenterology, Affiliated Foshan Hospital of Southern Medical University, Foshan 528000, China

***To whom correspondence should be addressed:**

Shunqian Wen; E-mail: wenshunqian0662@163.com

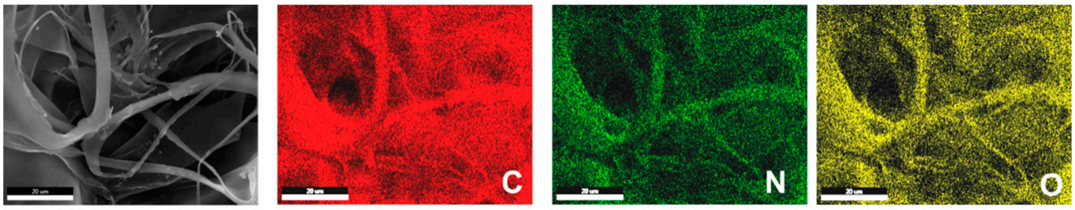

Figure S1. SEM photo and elemental mapping of PLEL hydrogel, scale bar = 20 μm.

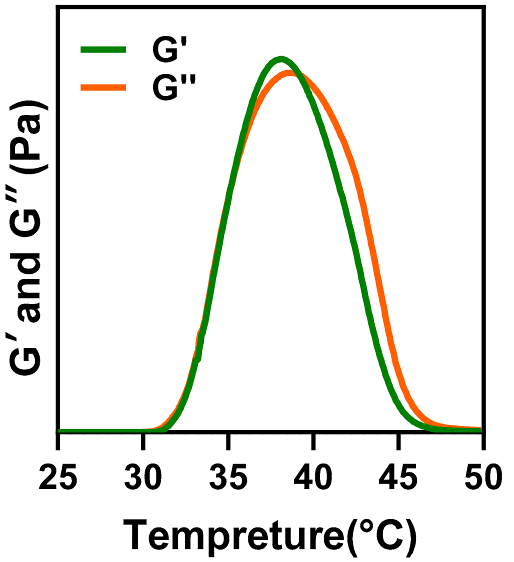

Figure S2. Rheological analysis of PLEL hydrogel.

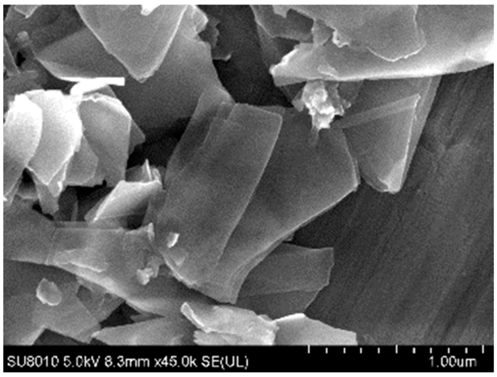

Figure S3. SEM photo of BP nanosheets.

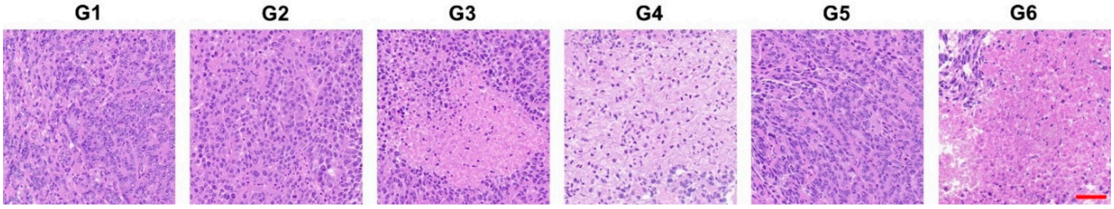

Figure S4. H&E staining of tumor slices from different treatment groups, scale bar = 50 μm.

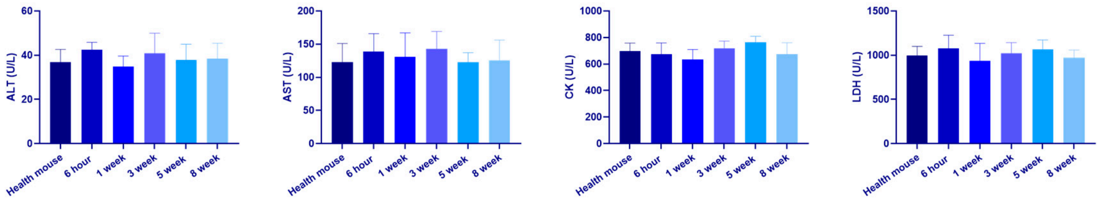

Figure S5. Biochemical indices of mice treated with RSL3/BP@PLEL at specific time intervals, all data are shown as mean ± S.D. (n = 3).
